# Supplementary material for: Boosting organic phosphorescence in adaptive host-guest materials by hyperconjugation
Source: Nat Commun. 2024 Apr 30;15:3660. doi: 10.1038/s41467-024-47992-0 (PMC11061117; doi:10.1038/s41467-024-47992-0)
Supplement: Supplementary file 1 — supplementary information [file 41467_2024_47992_MOESM1_ESM.pdf]

Supplementary Information

Boosting organic phosphorescence in adaptive host-guest materials by hyperconjugation

Huili Ma,[1] Lishun Fu,[1] Xiaokang Yao,[1] Xueyan Jiang,[1] Kaiqi Lv,[1] Qian Ma,[1] Huifang Shi,[1] Zhongfu An,[1*] Wei Huang[1,2*]

[1]*Key Laboratory of Flexible Electronics (KLOFE) and Institute of Advanced Materials (IAM), Nanjing Tech University (NanjingTech), Nanjing, China.*

[2]*Frontiers Science Center for Flexible Electronics (FSCFE), MIIT Key Laboratory of Flexible Electronics (KLoFE), Northwestern Polytechnical University, Xi'an, China.*

**Correspondence to: iamzfan@njtech.edu.cn; vc@nwpu.edu.cn*

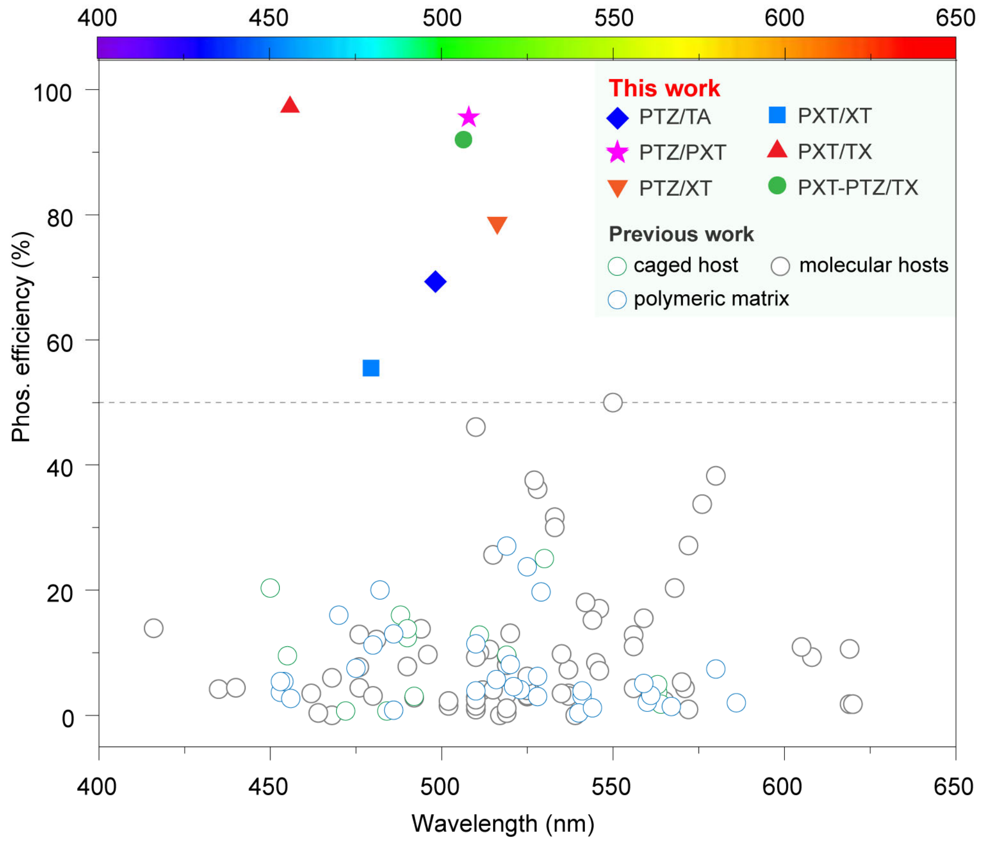

Supplementary Figure 1. Distribution showing phosphorescence emission peaks and efficiency of the heavy-atom-free host-guest systems under ambient conditions[9-36].

Supplementary Note 1 Hyperconjugation

Hyperconjugation is fundamentally a stabilizing interaction in which the electrons in certain σ bonds delocalize into an empty or partially occupied antibonding σ or π orbital (σ* or π*),[1,2] it is commonly invoked to explain the stability of alkyl substituted radicals and carbocations, represented by the σ→σ* and σ→π* hyperconjugative interaction[3,4]. On the basis of this concept, the other types of hyperconjugation, n→σ* and n→π* orbital interaction, were applied to study the stability of silicone polymers[5] and prtein strcuture[6,7]. From view of quantum mechanics, this hyperconjugation can be described by the second-order perturbative energies ($\Delta E_{ij}^{(2)}$) arising from the interaction of the occupied bonding orbital with the unoccupied antibonding orbital as seen in **Supplementary Fig. 2**, which can be evaluated by natural population analysis (NBO).[8]

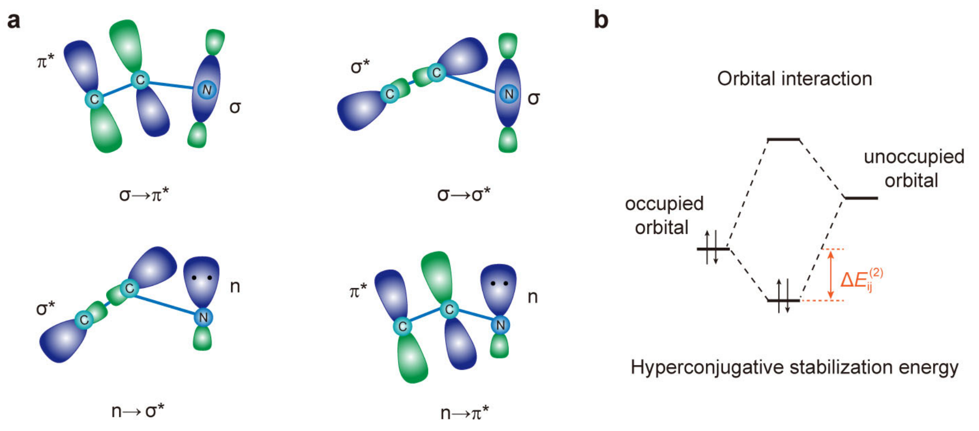

Supplementary Figure 2. Hyperconjugation. **a**, The types of n→π*, n→σ*, σ→π* and σ→σ* orbital interactions. **b**, The orbital interaction diagram showing the hyperconjugation stabilization energies ($\Delta E_{ij}^{(2)}$) arising from the interaction of the occupied bonding orbital with the unoccupied antibonding orbital.

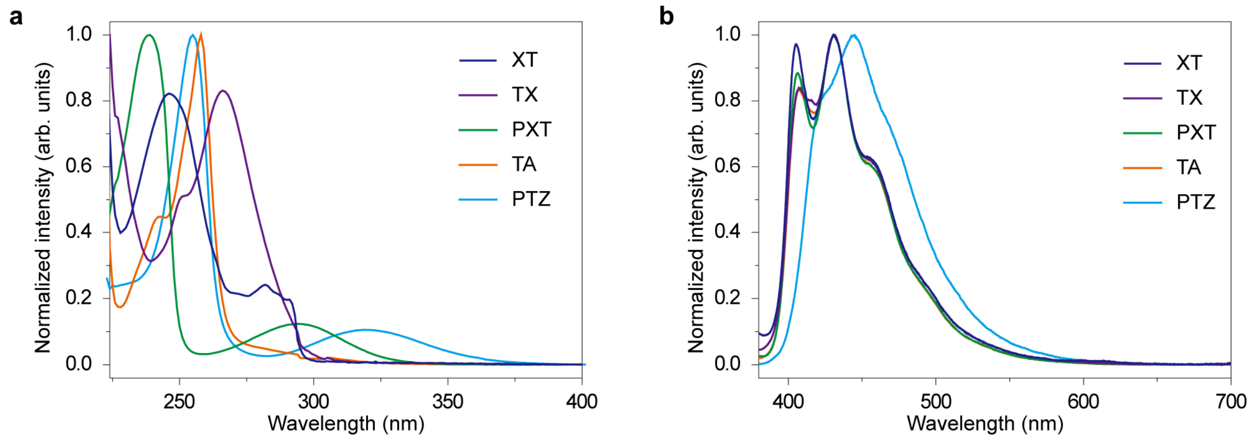

Supplementary Figure 3. Photophysical properties of the XT, TX, PXT, TA, and PTZ in dilute 2-mTHF solution (2×10^{-5} M) under ambient conditions. **a** Normalized UV absorption spectra. **b** Normalized steady-state PL spectra excited at 330 nm.

Supplementary Table 1. Fluorescence lifetimes of XT, TX, PXT, TA, and PTZ in dilute 2-mTHF solutions (2×10^{-5} M) under ambient conditions excited at 340 nm.

Molecules	XT	TX	PXT	TA	PTZ
Lifetime (ns)	1.11	1.30	1.25	1.27	0.97

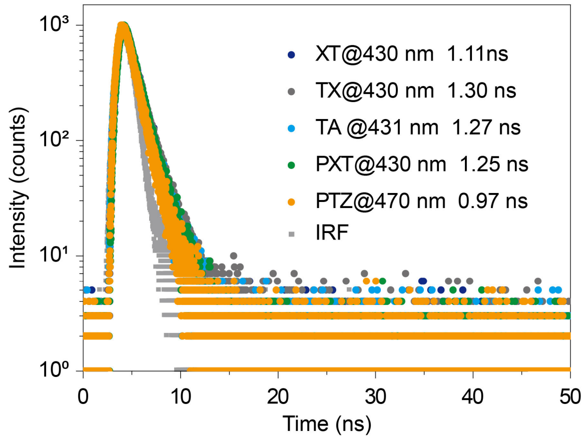

Supplementary Figure 4. Fluorescence lifetime decay profiles of dibenzo-heterocyclic analogues. Fluorescence lifetime decay profiles of XT, TX, PXT, TA, and PTZ in dilute 2-mTHF solutions (2×10^{-5} M) under ambient conditions excited at 340 nm.

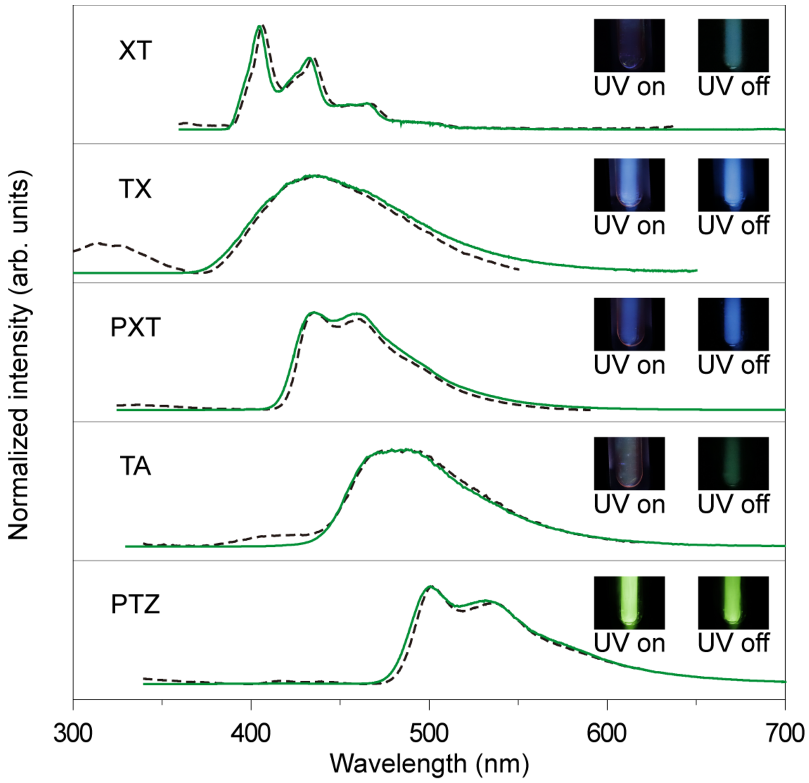

Supplementary Figure 5. Photoysical propterties for dibenzo-heterocyclic analogues. Normalized steady-state PL spectra (black dashed line) and phosphorescence spectra (green solid line) of XT, TX, PXT, TA, and PTZ in dilute 2-mTHF solution (2×10^{-5} M) under 77 K excited at 310 nm.

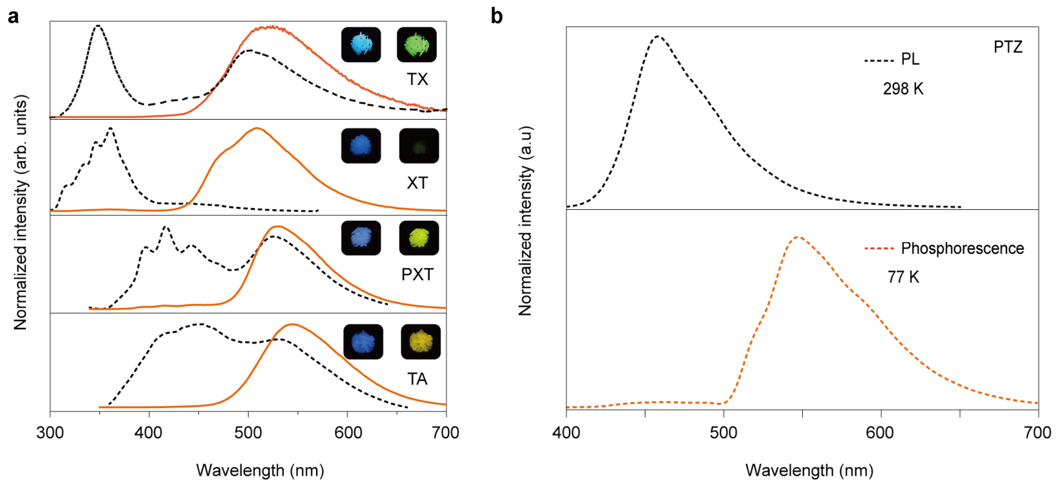

Supplementary Figure 6. Photophysical properties of the TX, XT, PXT, TA, and PTZ crystals. a Normalized steady-state PL (black dashed line) and phosphorescence spectra (orange solid line) of the TX, XT, PXT, and TA crystals under ambient conditions. **b** Normalized steady-state PL spectrum (black dashed line) of PTZ crystal under ambient conditions and the phosphorescence spectrum of PTZ at 77 K (orange dashed line) excited at 360 nm.

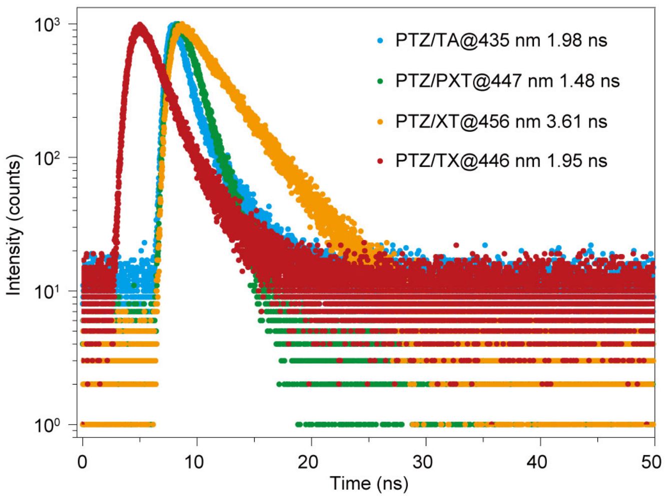

Supplementary Figure 7. Fluorescence lifetime decay profiles. Fluorescence lifetime decay profiles of the PTZ/TA, PTZ/PXT, PTZ/XT, and PTZ/TX mixed crsytals under ambient conditions excited at 340 nm.

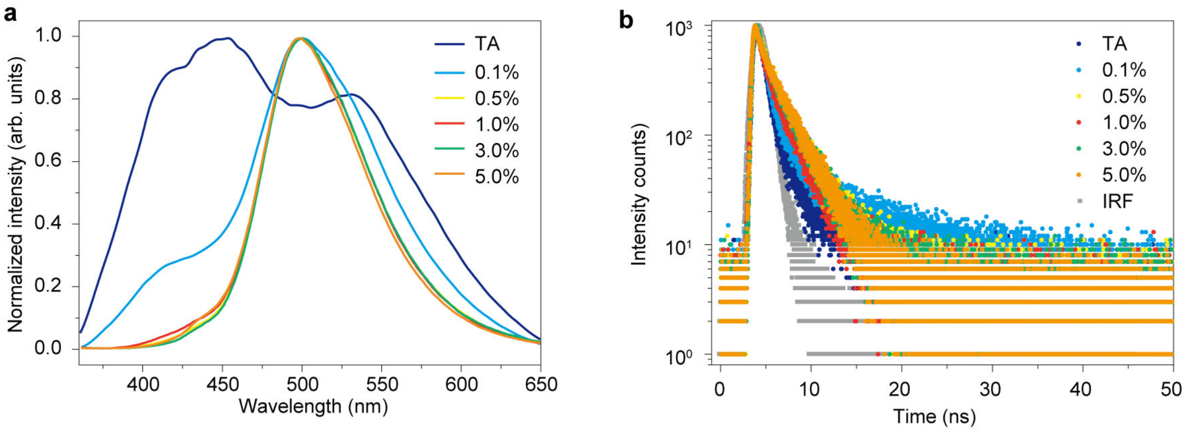

Supplementary Figure 8. Photophysical properties of the TA crystal and PTZ/TA with different doping concentrations from 0% to 5% (mol ratio) under ambient conditions. a Normalized steady-state PL spectra excited at 330 nm. **b** Fluorescence lifetime decay profiles monitoring 420 nm excited at 340 nm.

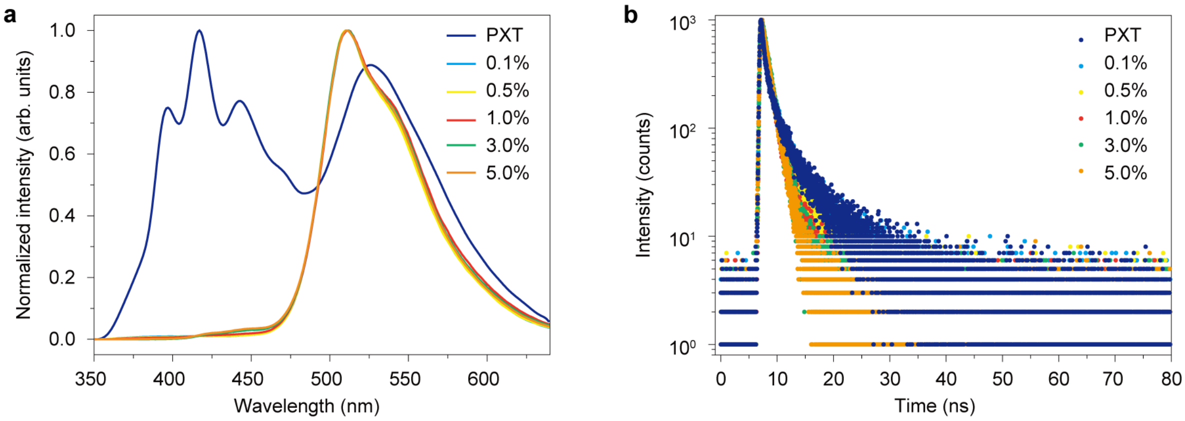

Supplementary Figure 9. Photophysical properties of the PXT crystal and PTZ/PXT with different doping concentrations from 0% to 5% (mol ratio) under ambient conditions. a Normalized steady-state PL spectra excited at 330 nm. **b** Fluorescence lifetime decay profiles monitoring 430 nm excited at 340 nm.

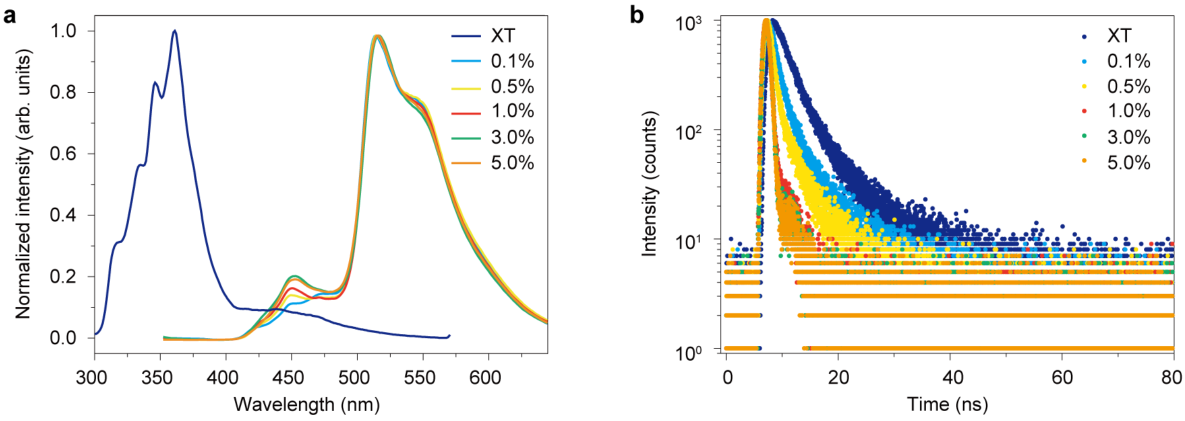

Supplementary Figure 10. Photophysical properties of the XT crystal and PTZ/XT with different doping concentrations from 0% to 5% (mol ratio) under ambient conditions. a Normalized steady-state PL spectra excited at 290 nm. **b** Fluorescence lifetime decay profiles mon itoring 360 nm excited at 280 nm.

Supplementary Table 2. Lifetimes and efficiency of the TA crystal and PTZ/TA with different doping concentrations from 0.1% to 5% (mol ratio) under ambient conditions.

Doping ratio (mol ratio)	Fluorescence lifetime (ns)	Phosphorescence lifetime (ms)		Phosphorescence efficiency (%)
		508 nm	530 nm	
TA	2.35	55.33	189.67	3.1
0.1%	3.18	130.43	96.68	1.9
0.5%	2.45	29.74	80.79	69.0
1.0%	2.19	49.46	43.67	35.9
3.0%	2.39	25.29	102.30	36.8
5.0%	2.42	14.66	20.54	36.0

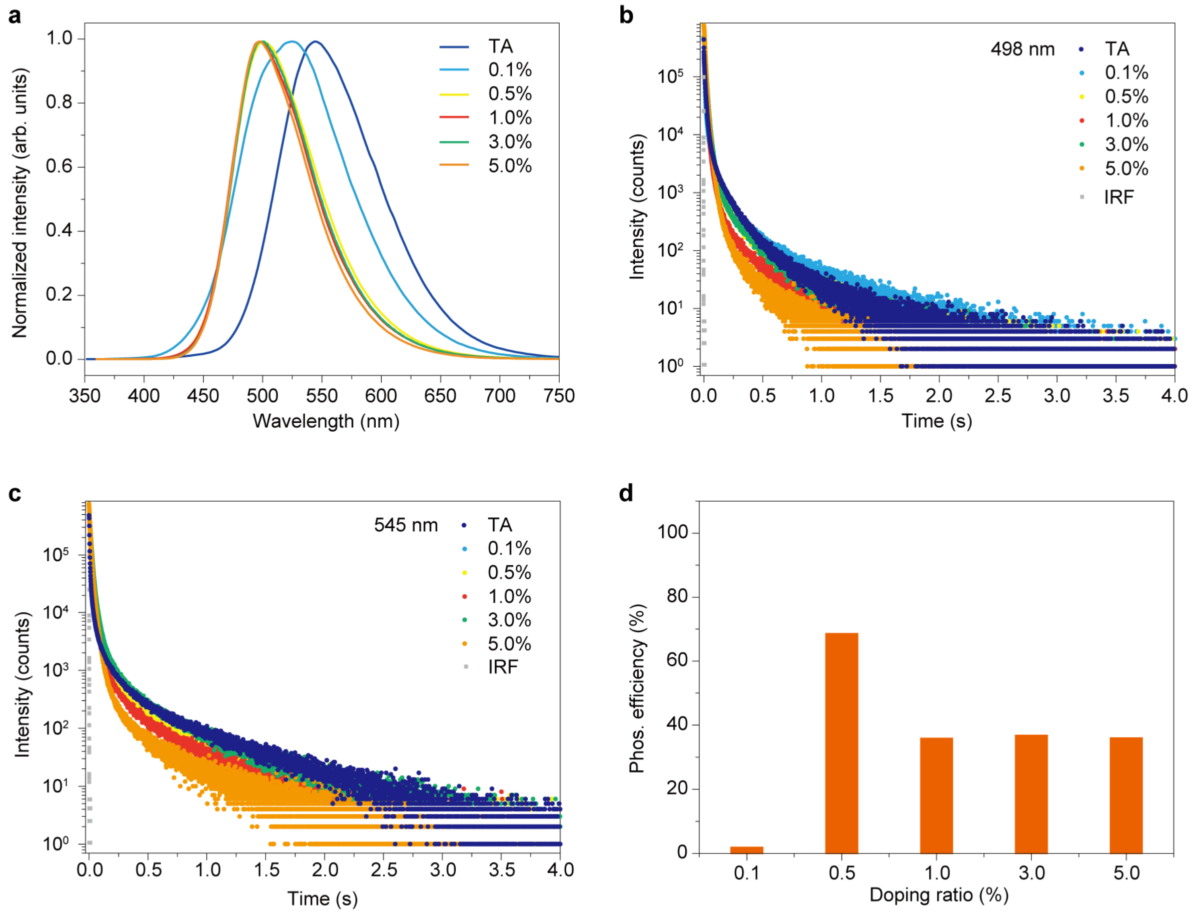

Supplementary Figure 11. Photophysical properties of the TA crystal and PTZ/TA with different doping concentrations from 0% to 5% (mol ratio) under ambient conditions. a Normalized phosphorescence spectra excited at 330 nm. **b**, **c** Phosphorescence lifetime decay profiles monitoring 498 nm and 545 nm excited at 330 nm. **d** Phosphorescnece quantum efficiency.

Supplementary Table 3. Lifetimes and efficiency of the PXT crystal and PTZ/PXT with different doping concentrations from 0.1% to 5% (mol ratio) under ambient conditions.

Doping ratio (mol ratio)	Fluorescence lifetime (ns)	Phosphorescence lifetime (ms)		Phosphorescence efficiency (%)
		508 nm	530 nm	
PXT	4.39	19.65	57.49	2.1
0.1%	3.09	43.36	31.71	88.2
0.5%	2.13	36.57	37.43	90.0
1.0%	1.65	44.81	32.80	96.8
3.0%	1.53	34.62	34.63	94.9
5.0%	1.41	32.03	32.14	93.9

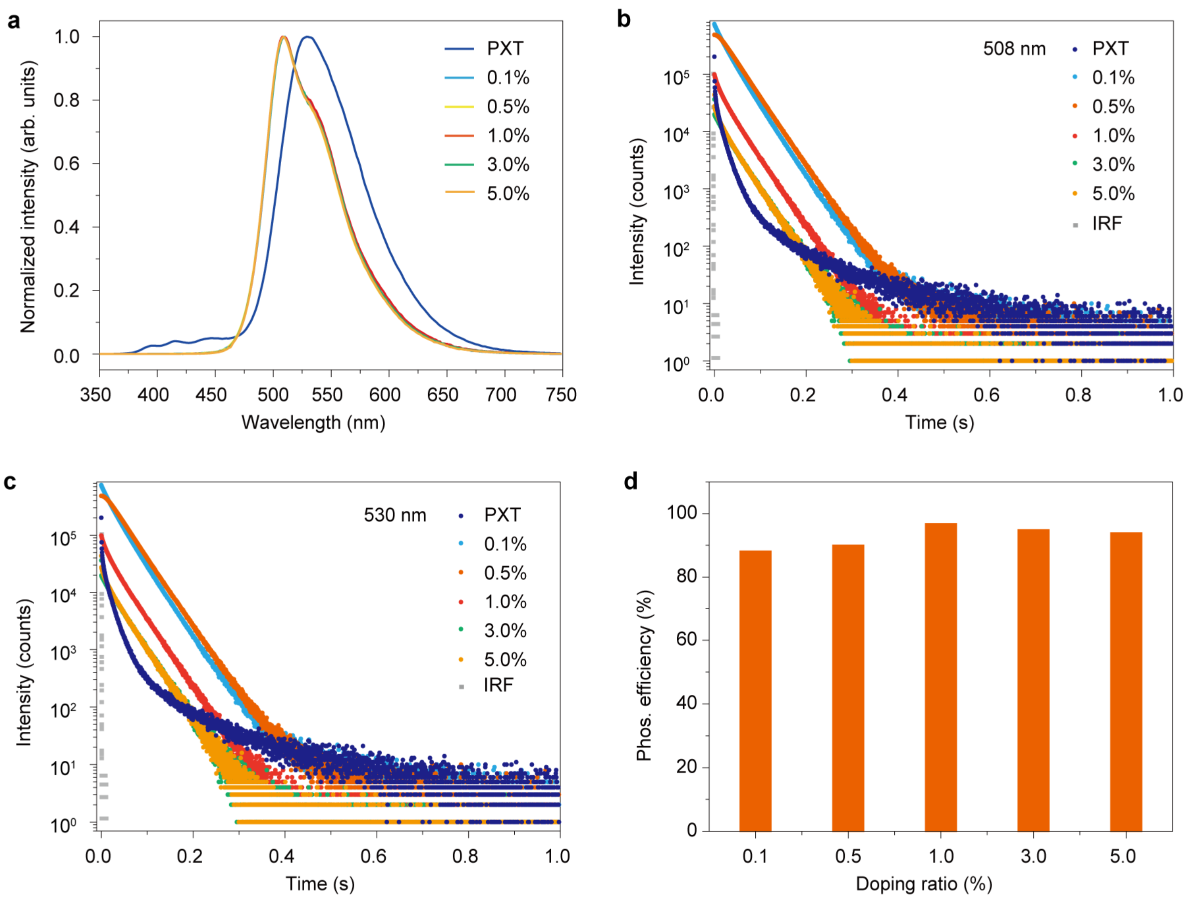

Supplementary Figure 12. Photophysical properties of the PTX crystal and PTZ/PXT with different doping concentrations from 0% to 5% (mol ration) under ambient conditions. a Normalized phosphorescence spectra excited at 330 nm. **b**, **c** Phosphorescence lifetime decay profiles monitoring 508 nm and 530 nm excited at 330 nm. **d** Phosphorescnece quantum efficiency.

Supplementary Table 4. Lifetimes and efficiency of XT crystal and PTZ/XT with different doping concentrations from 0.1% to 5% (mol ration) under ambient conditions.

Doping ratio (mol ratio)	Fluorescence lifetime (ns)	Phosphorescence lifetime (ms)		Phosphorescence efficiency (%)
		510 nm	515 nm	
XT	5.05	274.45	266.61	0.89
0.1%	3.38	48.91	48.45	49.2
0.5%	2.62	44.74	44.48	44.2
1.0%	1.42	41.41	41.44	78.6
3.0%	1.50	41.83	42.09	54.3
5.0%	1.57	41.26	40.78	53.3

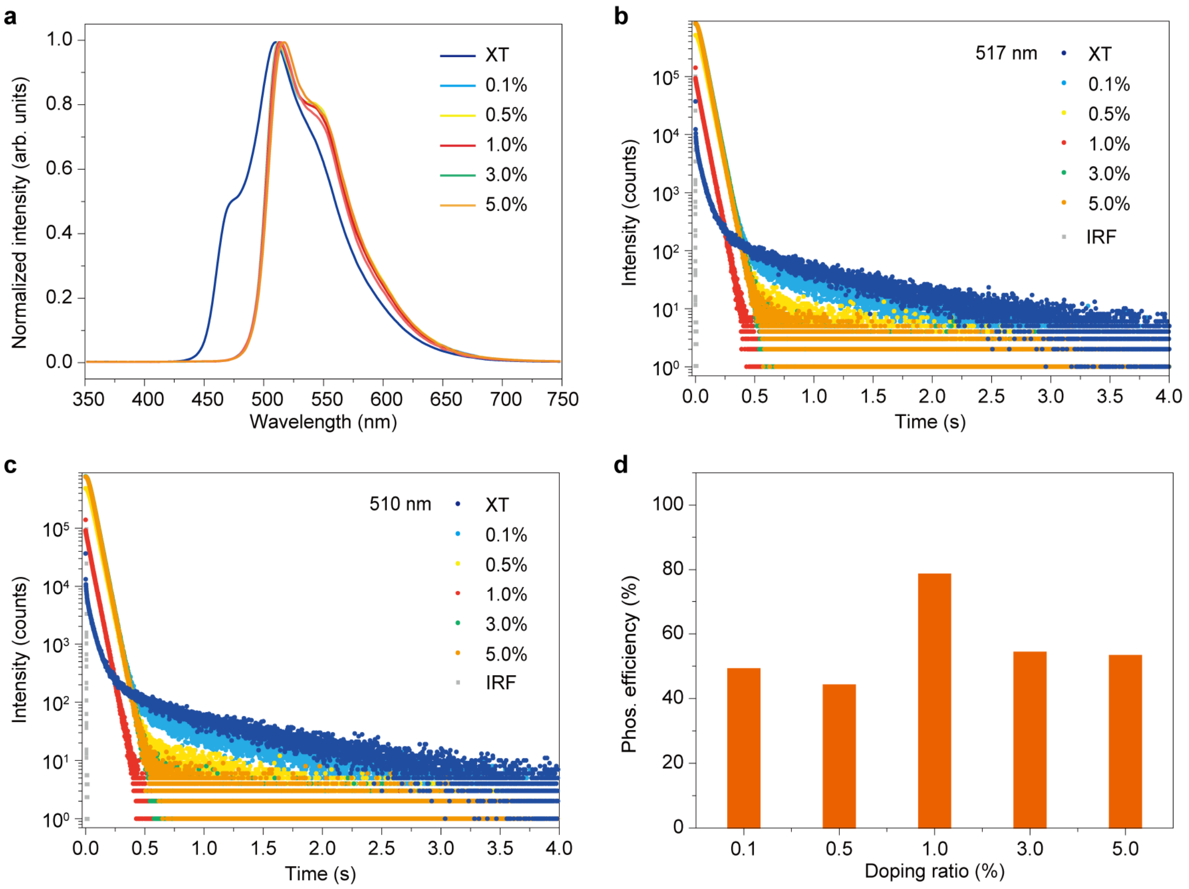

Supplementary Figure 13. Photophysical properties of the XT crystal and PTZ/XT with different doping concentrations from 0% to 5% (mol ratio) under ambient conditions. a Normalized phosphorescence spectra excited at 330 nm. **b**, **c** Phosphorescence lifetime decay profiles monitoring 517 nm and 510 nm excited at 330 nm. **d** Phosphorescence quantum efficiency.

Supplementary Table 5. Optimized quantum efficiency (QE) of PTZ/TA, PTZ/PXT, PTZ/XT, PXT/XT, PXT/TX, PTZ/TX and PT/XT under ambient conditions.

	Phosphorescence area	Total area	Phosphorescence ratio (%)	Total QE	Phos. QE
**PTZ/TA**	93.00	94.50	98.42	70.1	69.0
**PTZ/PXT**	76.08	76.71	99.18	97.6	96.8
**PTZ/XT**	78.92	82.43	95.74	82.1	78.6
**PXT/XT**	70.60	73.44	96.15	57.1	54.9
**PXT/TX**	81.28	82.18	98.90	100.0	98.9
**PTZ/TX**	92.55	94.75	97.68	86.3	84.3
**PT/XT**	112.03	116.29	96.34	95.5	92.0

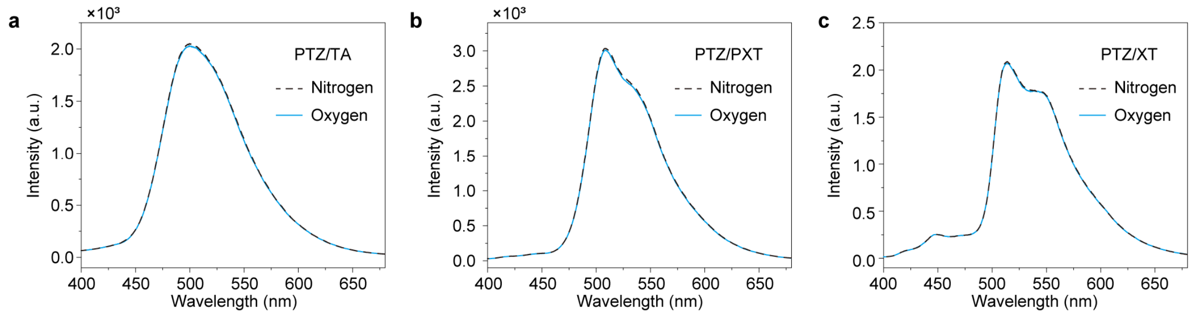

Supplementary Figure 14. The phosphorescence spectra of the adaptive host-guest materials under nitrogen and oxygen enviroments. a PTZ/TA. **b** PTZ/PXT. **c** PTZ/XT.

Supplementary Table 6. The structural variations of host/guest molecules in the QM region from PTZ/TA, PTZ/PXT to PTZ/XT based on S_0-geometry.

Host	$\Delta\theta_{PTZ}$	PTZ/G	H1	H2	$\Delta\theta_{H1}$	$\Delta\theta_{H2}$	$\Delta\theta_{H1\text{-}G}$	$\Delta\theta_{H2\text{-}G}$
TA	18°	137.8°	130.0°	128.6°	1.9°	0.5°	7.8°	9.2°
PXT	8.6°	147.2°	148.6°	148.5°	2.9°	2.8°	1.4°	1.3°
XT	22.0°	177.8°	170.2°	173.2°	4.1°	1.1°	7.6°	4.6°

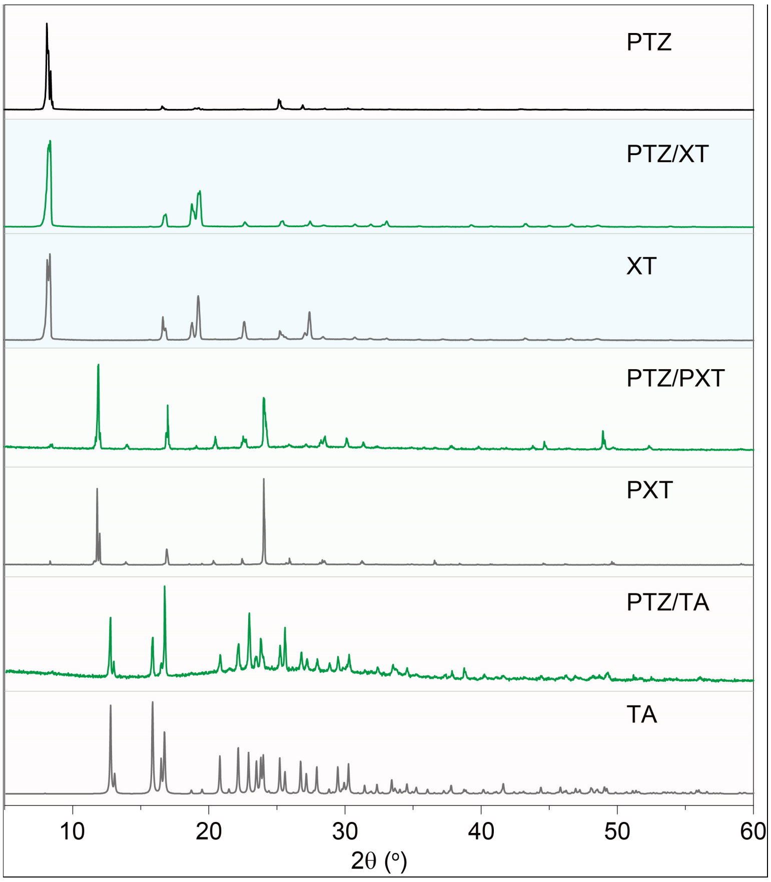

Supplementary Figure 15. Normalized XRD spectra for host single crystal and mixed crystals. The order from top to bottom is PTZ, PTZ/XT, XT, PTZ/PXT, PXT, PTZ/TA, and TA.

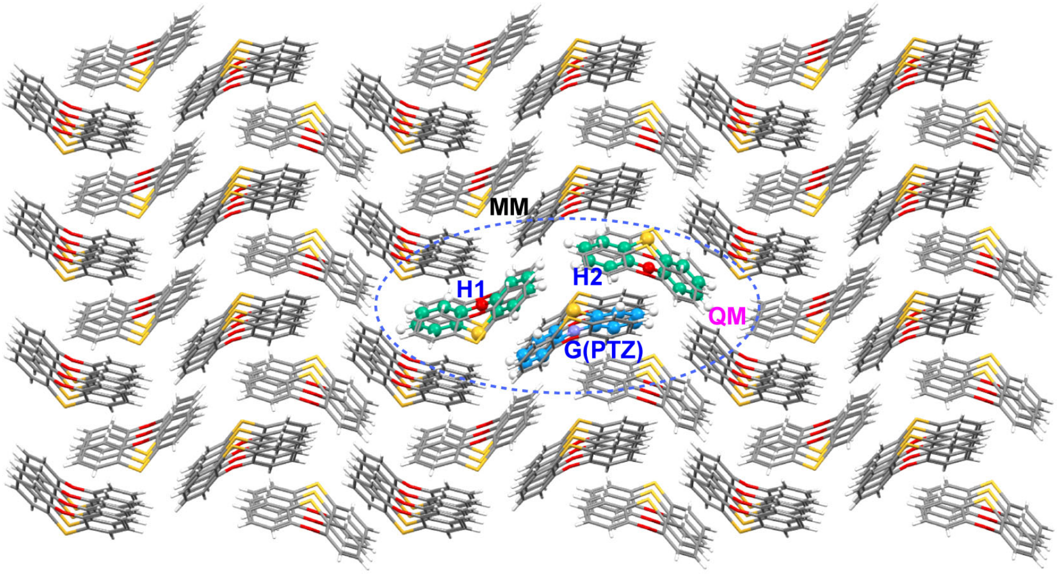

Supplementary Figure 16. Set up of QM/MM model. Taking PTZ/PXT as an example, one guest PTZ (G) plus two host molecules (H1 and H2) are defined as QM region, and the remains are chosen as fixed MM molecules.

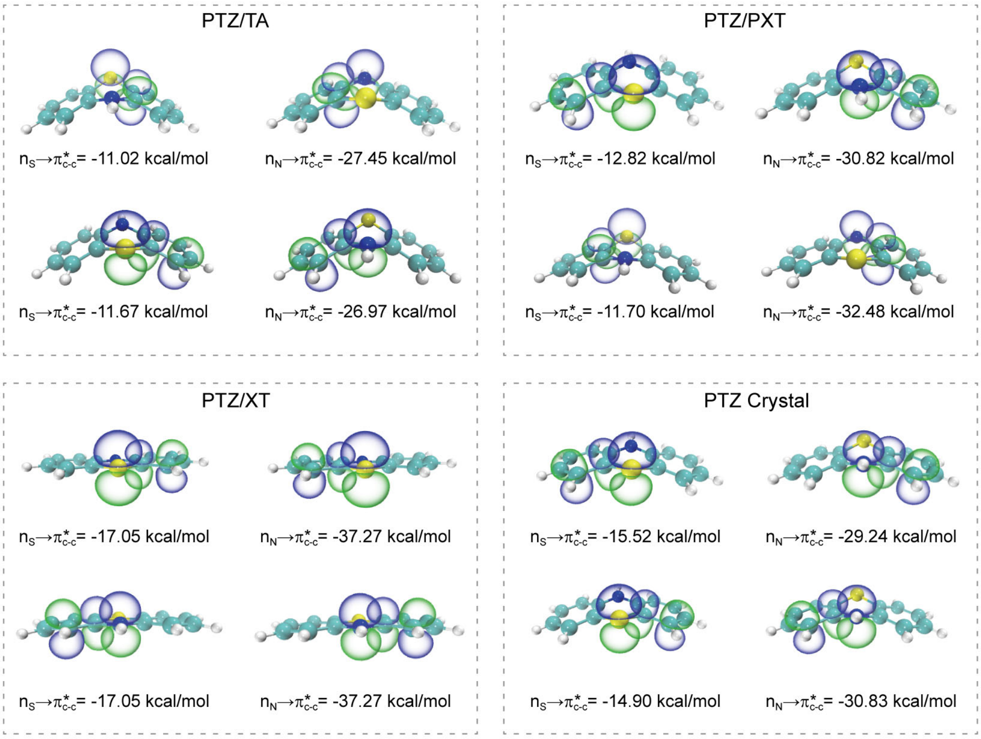

Supplementary Figure 17. Visualization of hyperconjugative stabilization energy. Vicinal π-type $n_X \rightarrow \pi^*_{C\text{-}C}$ (X=S/N) hyperconjugative stabilization energy ($E^{(2)}$) for PTZ in the PTZ/TA, PTZ/PXT, and PTZ/XT systems and PTZ crystal.

Supplementary Table 7. The conformational reorganization energy (λ_{CRE}) for PTZ-doping systems, compared to the PTZ crystal. The λ_{CRE} represents the energy gap between PTZ guest in host matrix and the single-crystal.

	PTZ/TA	PTZ/PXT	PTZ/XT
λ_{CRE} (kcal/mol)	1.148	0.484	2.135

Supplementary Table 8. The largest single values of vicinal $n_X \rightarrow \sigma^*_{C\text{-}C}$ and $n_X \rightarrow \pi^*_{C\text{-}C}$ (X=S/N) hyperconjugative stabilization energies ($E^{(2)}$, kcal/mol) for PTZ in PTZ/TA, PTZ/PXT and PTZ/XT systems and PTZ crystal.

	$n_S \rightarrow \sigma^*_{C\text{-}C}$	$n_S \rightarrow \pi^*_{C\text{-}C}$	$n_N \rightarrow \sigma^*_{C\text{-}C}$	$n_N \rightarrow \pi^*_{C\text{-}C}$
PTZ/TA	-3.45	-11.67	-1.50	-27.45
PTZ/PXT	-3.54	-12.82	-0.85	-32.48
PTZ/XT	-4.19	-17.05	-	-37.27
PTZ (crystal)	-4.17	-15.52	-1.73	-30.83

Supplementary Table 9. EDA for the intermolecular interactions energy (E_{int}) between guest PTZ and host molecules, including electrostatic (E_{ele}), repulsion (E_{rep}), and dispersion (E_{disp}) energies. The unit is kcal/mol.

	E_{ele}	E_{rep}	E_{disp}	E_{int}
PTZ/TA	-3.42	47.26	-96.17	-52.33
PTZ/PXT	-4.03	39.65	-75.42	-39.80
PTZ/XT	-7.30	59.80	-106.16	-53.66

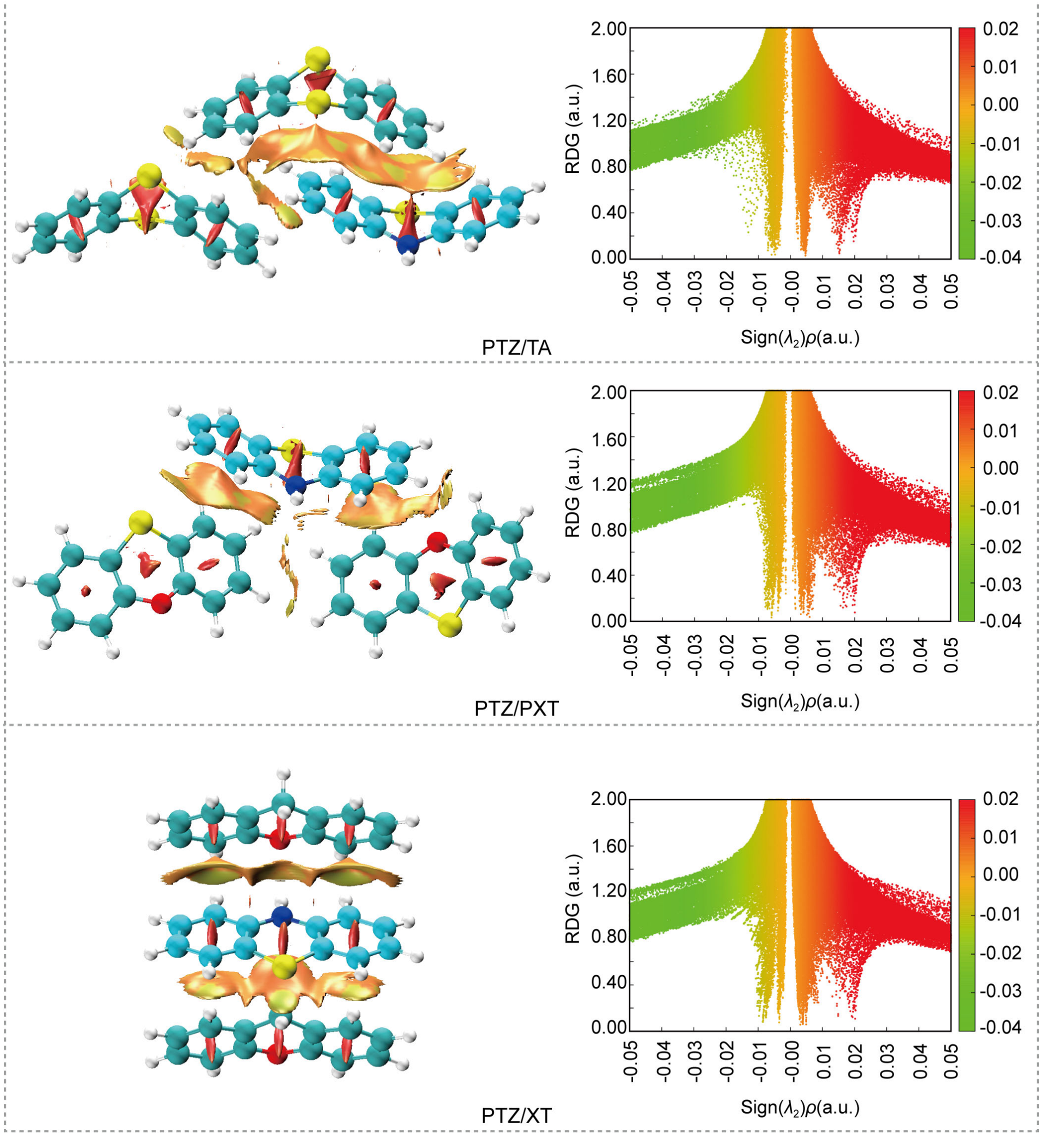

Supplementary Figure 18. Visualization of intermolecular interactions. Calculated reduced density gradient isosurfaces (isovalue= 0.70, left) and scatter graphs (right) for QM molecules in PTZ/TA, PTZ/PXT, and PTZ/XT.

Supplementary Table 10. Photophysical parameters of PTZ/TA, PTZ/PXT, PTZ/XT, PXT/XT, PXT/TX and PTZ/TX under ambient conditions.

	Φ_{PL} (%)	Φ_{P} (%)	Φ_{isc}[a)] (%)	τ_{p} (ms)	k_p (s^{-1}) [b)]	k_{nr} (s^{-1}) [c)]
PXT/TX	100.0	**98.9**	98.9	47.91	20.87	**0.00**
PTZ/PXT	97.6	**96.8**	99.2	44.81	21.78	**0.54**
PTZ/TX	86.3	**84.3**	98.0	39.50	21.78	**3.34**
PTZ/XT	82.1	**78.6**	96.5	41.44	19.66	**4.48**
PTZ/TA	70.1	**69.0**	99.1	29.74	23.41	**10.21**
PXT/XT	57.1	**54.9**	97.8	22.48	24.97	**19.51**

a)Φ_{isc} = 1- Φ_F -Φ_{ic} ≈ 1- Φ_{PL} + Φ_P; b)k_p = $\Phi_P/(\Phi_{isc} \times \tau_p)$; c)$k_{nr}$ = $1/\tau_p$ - k_p

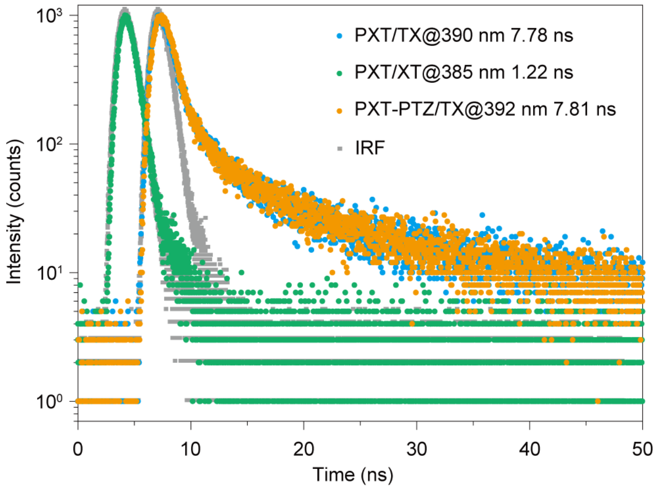

Supplementary Figure 19. Fluorescence lifetime decay profiles. Fluorescence lifetime decay profiles of PXT/TX, PXT/XT, and PXT-PTZ/TX mixed crystals under ambient conditions excited at 340 nm.

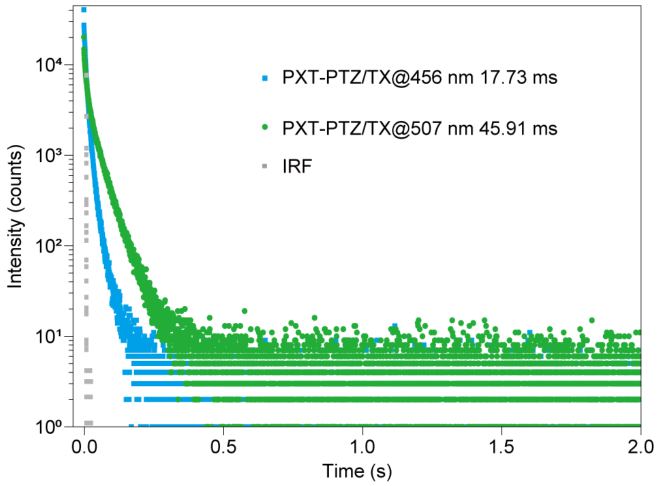

Supplementary Figure 20. Phosphorescence lifetime profiles. Phosphorescence lifetime profiles of the PT/TX solids monitoring 456 and 507 nm under ambient conditions excited at 330 nm.

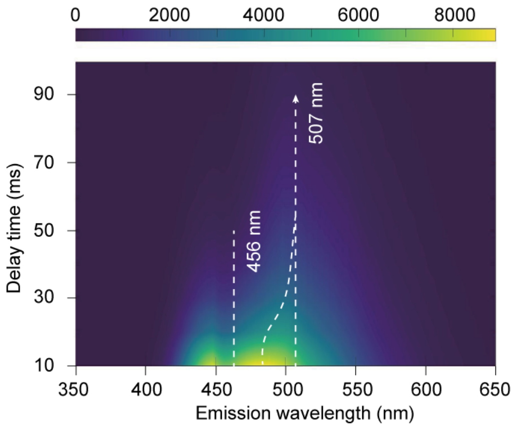

Supplementary Figure 21. Time-resolved emission spectra of ternary host-guest system. Transient photoluminescence decay image of the PT/TX excited at 310 nm.

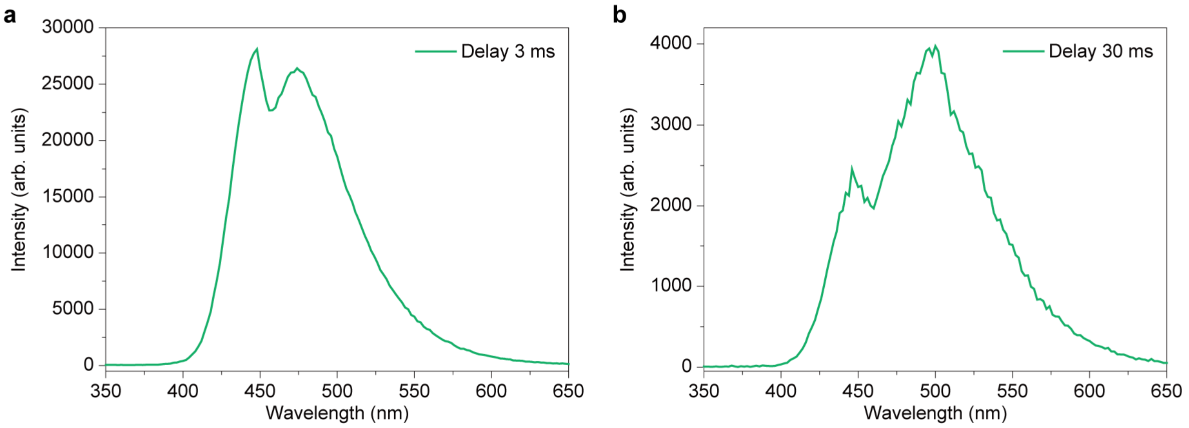

Supplementary Figure 22. Time-resolved photoluminance spectra of the PT/TX solids under ambient conditions excited at 330 nm. a, b After 3 ms and 30 ms delay time, respectively.

Supplementary Note 2 Calculation of the phosphorescence quantum efficiency

In our manuscript, the phosphorescence quantum efficiency (QE) was collected as follows. The total photoluminescence quantum yield was first obtained using an absolute photoluminescence (PL) quantum yield spectrometer (HAMAMATSU C11347) with an integrating sphere under ambient conditions. The phosphorescence quantum yields of the compounds were obtained from the following equation:

$$\phi_{\mathrm{phos}} = \frac{B}{A+B} \times \phi_{\mathrm{PL}}$$

where A and B represent the integral areas of fluorescence and phosphorescence spectra, respectively. The phosphorescence was separated from the total PL spectrum based on the phosphorescence spectrum (**Supplementary Fig. 23**).

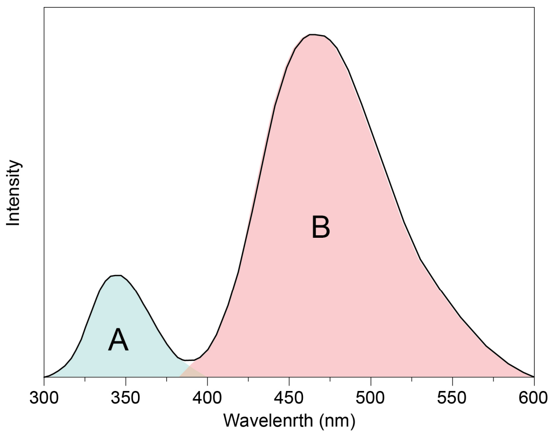

Supplementary Figure 23. Calculation of the phosphorescence quantum efficiency. Schematic diagram of integral areas of fluorescence (A) and phosphorescence (B) for quantum yield calculation.

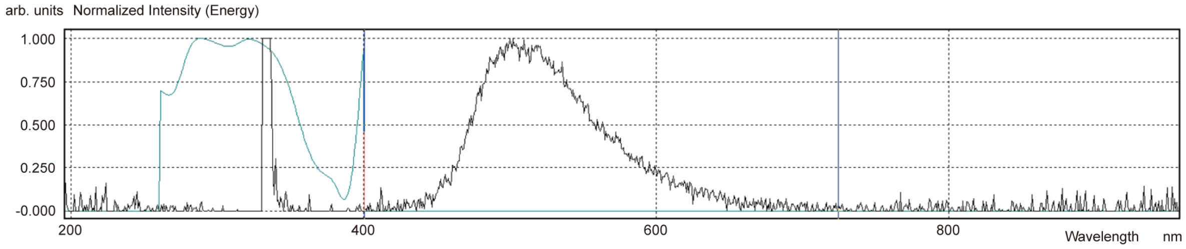

Cursor1-2	Cursor3-4: 400.23-729.59(nm)	QuantumYield	Abs	Exc. Correction coeff.	PeakWavelength	PeakCounts	Peak FWHM	PeakWavelength	PeakCounts	Peak FWHM
80672670	---	---	---	0.463	319.93	41011.23	5.80	401.74	35.11	152.68
73696558	4889217	0.701	0.086	---	320.69	37365.24	5.80	510.89	106.09	94.04

Supplementary Figure 24. Original data of the PTZ/TA phosphor for phosphorescence efficiency calculation.

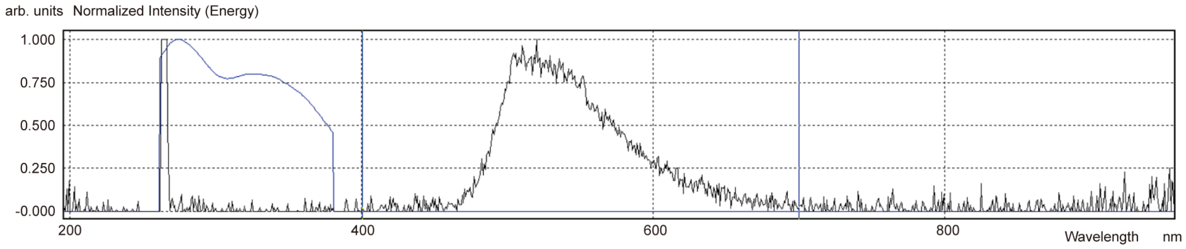

Cursor1-2	Cursor3-4: 400.23-729.59(nm)	QuantumYield	Abs	Exc. Correction coeff.	PeakWavelength	PeakCounts	Peak FWHM	PeakWavelength	PeakCounts	Peak FWHM
23548937	---	---	---	0.202	280.38	12874.37	6.25	401.74	15.52	202.88
18051456	5363125	0.976	0.233	---	281.14	9909.38	6.20	508.64	124.80	85.74

Supplementary Figure 25. Original data of the PTZ/PXT phosphor for phosphorescence efficiency calculation.

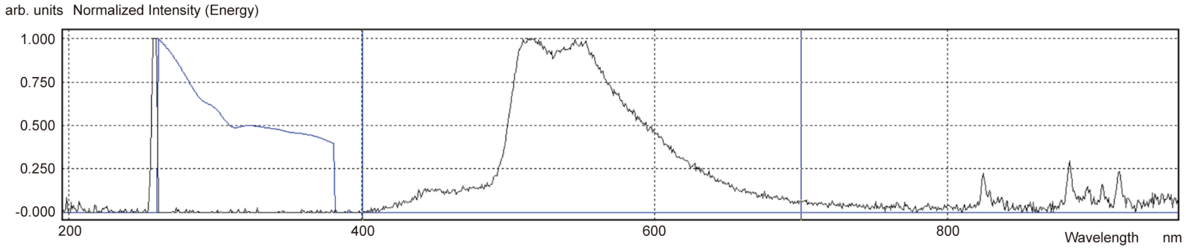

Cursor1-2	Cursor3-4: 400.23-729.59(nm)	QuantumYield	Abs	Exc. Correction coeff.	PeakWavelength	PeakCounts	Peak FWHM	PeakWavelength	PeakCounts	Peak FWHM
32173728	---	---	---	0.127	270.48	18006.16	6.29	405.52	25.96	83.18
18967815	10838424	0.821	0.410	---	270.48	10651.02	6.27	513.89	237.99	95.36

Supplementary Figure 26. Original data of the PTZ/XT phosphor for phosphorescence efficiency calculation.

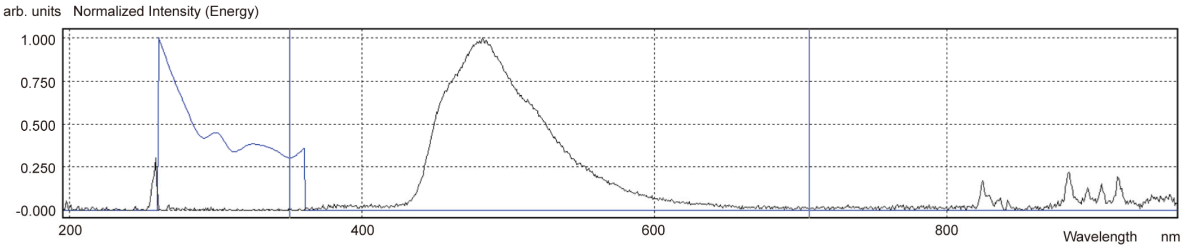

Cursor1-2	Cursor3-4: 350.28-700.05(nm)	QuantumYield	Abs	Exc. Correction coeff.	PeakWavelength	PeakCounts	Peak FWHM	PeakWavelength	PeakCounts	Peak FWHM
31658062	---	---	---	0.156	270.48	17839.94	6.24	350.28	112.70	119.28
12633767	10856448	0.571	0.601	---	270.48	7094.50	6.26	483.12	351.47	77.43

Supplementary Figure 27. Original data of the PXT/XT phosphor for phosphorescence efficiency calculation.

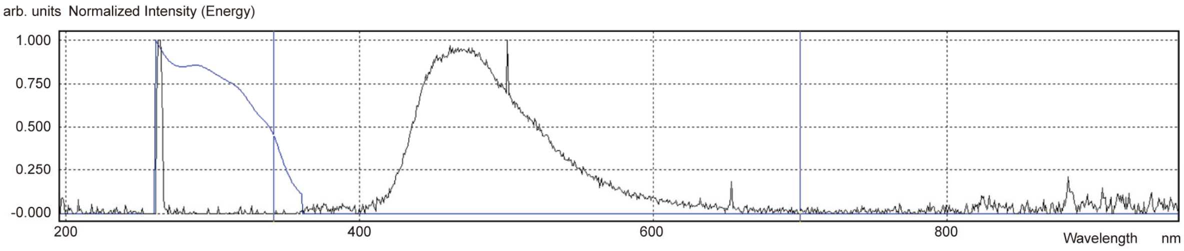

Cursor1-2	Cursor3-4: 341.18-700.05(nm)	QuantumYield	Abs	Exc. Correction coeff.	PeakWavelength	PeakCounts	Peak FWHM	PeakWavelength	PeakCounts	Peak FWHM
136083867	---	---	---	0.567	310.05	68019.04	6.12	341.18	378.09	76.03
104174819	31923910	1.000	0.234	---	310.81	52313.29	6.07	468.83	765.53	89.00

Supplementary Figure 28. Original data of the PXT/TX phosphor for phosphorescence efficiency calculation.

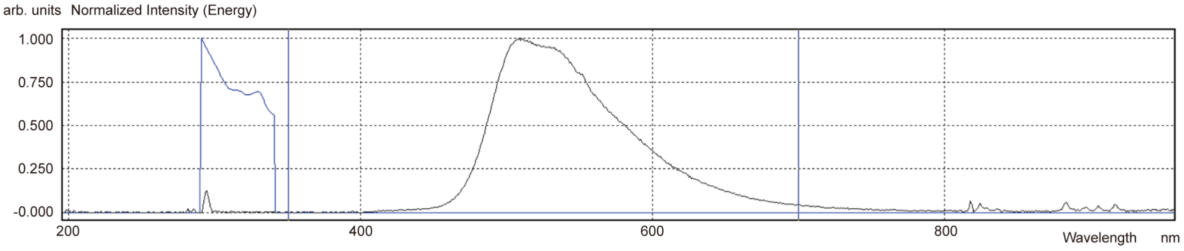

Cursor1-2	Cursor3-4: 350.28-700.05(nm)	QuantumYield	Abs	Exc. Correction coeff.	PeakWavelength	PeakCounts	Peak FWHM	PeakWavelength	PeakCounts	Peak FWHM
65613224	---	---	---	0.568	305.49	30745.27	6.28	350.28	158.62	131.61
13559056	44912978	0.863	0.793	---	305.49	6235.12	6.41	510.14	941.07	94.93

Supplementary Figure 29. Original data of the PTZ/TX phosphor for phosphorescence efficiency calculation.

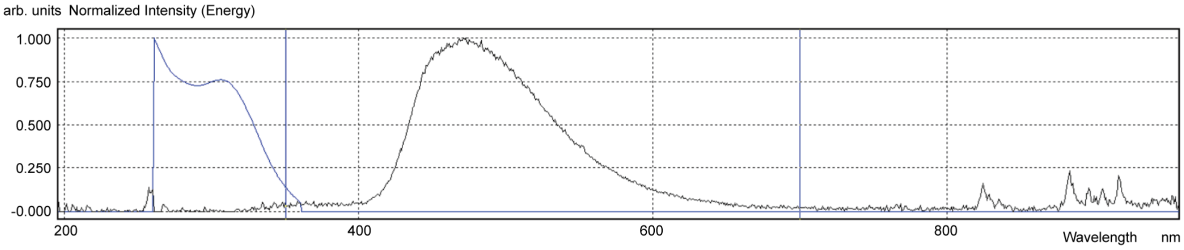

Cursor1-2	Cursor3-4: 350.28-700.05(nm)	QuantumYield	Abs	Exc. Correction coeff.	PeakWavelength	PeakCounts	Peak FWHM	PeakWavelength	PeakCounts	Peak FWHM
104697486	---	---	---	0.478	300.93	53119.46	6.21	350.28	180.84	98.27
57094094	45454723	0.955	0.455	---	300.17	29330.42	6.12	481.61	867.66	111.42

Supplementary Figure 30. Original data of the PT/XT phosphor for phosphorescence efficiency calculation.

Supplementary References

1. Reed, A. E., Curtiss, L. A. & Weinhold, F. Intermolecular interactions from a natural bond orbital, donor-acceptor viewpoint. *Chem. Rev.* **88**, 899-926 (1988).
2. Alabugin, I. V., Gilmore, K. M. & Peterson, P. W. Hyperconjugation. *Wiley Interdiscip. Rev.: Comput. Mol. Sci.* **1**, 109-141 (2011).
3. Pophristic, V. & Goodman, L. Hyperconjugation not steric repulsion leads to the staggered structure of ethane. *Nature* **411**, 565-568 (2001).
4. Dajnowicz, S. *et al.* Hyperconjugation promotes catalysis in a pyridoxal 5'-phosphate-dependent enzyme. *ACS Catal.* **8**, 6733- 6737 (2018).
5. Weinhold, F. & West, R. Hyperconjugative interactions in permethylated siloxanes and ethers: the nature of the SiO nond. *J. Am. Chem. Soc.*, **135**, 5762-5767 (2013).
6. Bartlett, G., Choudhary, A., Raines, R.*et al.* $n \rightarrow \pi^*$ interactions in proteins. *Nat. Chem. Biol.* **6,** 615-620 (2010).
7. Imani, Z. *et al.* Conformation control through concurrent N-H···S and N-H···O-C hydrogen bonding and hyperconjugation effects. *Chemi. Sci.*, 11, 9191-9197 (2020).
8. Reed, A. E., Weinstock, R. B. & Weinhold, F. Natural population analysis. *J. Chem. Phys.* **83**, 735-746 (1985).
9. Hirata, S. Recent advances in materials with room temperature phosphorescence: photophysics for triplet exciton stabilization. *Adv. Opt. Mater.* **5**, 1700116 (2017).
10. Zhang, Z. et al. A Synergistic enhancement strategy for realizing ultralong and efficient room-temperature phosphorescence. *Angew. Chem. Int. Ed.* **59**, 18748-18754 (2020).
11. Yong, G., Zhang, X. & She, W. Phosphorescence enhancement of organic dyes by forming b-cyclodextrin inclusion complexes: Color tunable emissive materials. *Dyes Pigm.* **97**, 65-70 (2013).
12. Zhao, Y., Yong, G., Zhang, X. & Zhang, B. Reversibly photoswitchable dual-color (blue ↔ green) phosphorescence from ß-cyclodextrin inclusion complex materials. *Dyes Pigm.* **101**, 172-178 (2014).
13. Mieno, H., Kabe, R. & Adachi, C. Reversible control of triplet dynamics in metalorganic framework-entrapped organic emitters via external gases. *Commun. Chem.* **1**, 27 (2018).
14. Lei, Y. et al. Wide-range color-tunable ultralong organic phosphorescence materials for printable and writable security inks. *Angew. Chem. Int. Ed.* **59**, 16054-16060 (2020).
15. Wang, Y. et al. Förster resonance energy transfer: an efficient way to develop stimulus-responsive room-temperature phosphorescence materials and their applications. *Matter* **3**, 449-463 (2020).
16. Notsuka, N., Kabe, R., Goushi, K. & Adachi, C. Confinement of long-lived triplet excitons in organic semiconducting host-guest systems. *Adv. Funct. Mater.* ***27***, 1703902 (2017).
17. Lei, Y. et al. Revealing insight into long-lived room-temperature phosphorescence of host-guest systems. *J. Phys. Chem. Lett.* **10**, 6019-6025 (2019).

18. Su, Y. et al. Excitation-dependent long-life luminescent polymeric systems under ambient conditions. *Angew. Chem. Int. Ed.* **59**, 9967-9971 (2020).
19. Salas Redondo, C. *et al.* Interplay of fluorescence and phosphorescence in organic biluminescent emitters. *J. Phys. Chem. C.* **121**, 14946-14953 (2017).
20. Wu, H. et al. Achieving amorphous ultralong room temperature phosphorescence by coassembling planar small organic molecules with polyvinyl alcohol. *Adv. Funct. Mater.* **29**, 1807243 (2019).
21. Xiao, L. et al. Highly efficient room-temperature phosphorescence from halogen-bonding-assisted doped organic crystals. *J. Phys. Chem. A.* **121**, 8652-8658 (2017).
22. Louis, M. et al. Biluminescence under ambient conditions: water-soluble organic emitter in high-oxygen-barrier polymer. *Adv. Optical Mater.* **8**, 2000427 (2020).
23. Louis, M. et al. Blue-light-absorbing thin films showing ultralong room-temperature phosphorescence. *Adv. Mater.* **31**, 1807887 (2019).
24. Thomas, H. *et al.* Aromatic phosphonates: a novel group of emitters showing blue ultralong room temperature phosphorescence. *Adv Mater.* **32**, 2000880 (2020).
25. Jõgela, J., Uri, A., Pålsson, L. & Enkvist E. Almost complete radiationless energy transfer from excited triplet state of a dim phosphor to a covalently linked adjacent fluorescent dye in purely organic tandem luminophores doped into PVA matrix. *J. Mater. Chem. C.* **7**, 6571 (2019).
26. Sn, Y. et al. Ultralong room temperature phosphorescence from amorphous organic materials toward confidential information encryption and decryption. *Sci. Adv.* **4**, eaas9732 (2018).
27. Bhattacharjee, I. & Hirata, S. Highly efficient persistent room-temperature phosphorescence from heavy atom-free molecules triggered by hidden long phosphorescent antenna. *Adv. Mater.* **32**, 2001348 (2020).
28. Hirata, S. Intrinsic analysis of radiative and room-temperature nonradiative processes based on triplet state intramolecular vibrations of heavy atom-free conjugated molecules toward efficient persistent room-temperature phosphorescence. *J. Phys. Chem. Lett.* **9**, 4251-4259 (2018).
29. Liu, X. et al. Influence of guest/host morphology on room temperature phosphorescence properties of pure organic doped systems. *J. Phys. Chem. Lett.* **12**, 7357-7364 (2021).
30. Yang, J. et al. Achieving efficient phosphorescence and mechanoluminescence in organic host-guest system by energy transfer. *Adv. Funct. Mater.* **31**, 2108072 (2021).
31. Wang, D. et al. Excitation-dependent triplet–singlet intensity from organic host–guest materials: tunable color, white-light emission, and room-temperature phosphorescence. *J. Phys. Chem. Lett.* **12**, 1814-1821 (2021).
32. Tian, Y.et al. multistage stimulus-responsive room temperature phosphorescence based on host–guest doping systems. *Angew. Chem. Int. Ed.* **60**, 20259-20263 (2021).

33. Li, M. et al. Boosting purely organic room-temperature phosphorescence performance through a host-guest strategy. *Chemi. Sci.* **12**, 13580-13587 (2021).

34. Zhang, Y. et al. Large-area, flexible, transparent, and long-lived polymer-based phosphorescence films. *J. Am. Chem. Soc.* **143**, 13675-13685 (2021).

35. Li, D. et al. Completely aqueous processable stimulus responsive organic room temperature phosphorescence materials with tunable afterglow color. *Nat. Commun.* **13**, 347 (2022).

36. Chanmungkalakul, S. et al. A descriptor for accurate predictions of host molecules enabling ultralong room-temperature phosphorescence in guest emitters. *Angew. Chem. Int. Ed.* e202200546 (2022).
