# Supplementary material for: Boosting organic phosphorescence in adaptive host-guest materials by hyperconjugation
Source: Nat Commun. 2024 Apr 30;15:3660. doi: 10.1038/s41467-024-47992-0 (PMC11061117; doi:10.1038/s41467-024-47992-0)
Supplement: Supplementary file 3 — Reporting Summary [file 41467_2024_47992_MOESM3_ESM.pdf]

nature portfolio

Corresponding author(s): Zhongfu An, Wei Huang

Last updated by author(s): Mar 17, 2024



# Reporting Summary

Nature Portfolio wishes to improve the reproducibility of the work that we publish. This form provides structure for consistency and transparency in reporting. For further information on Nature Portfolio policies, see our Editorial Policies and the Editorial Policy Checklist.

## Statistics

For all statistical analyses, confirm that the following items are present in the figure legend, table legend, main text, or Methods section.

| n/a | Confirmed | |
|---|---|---|
| x | | The exact sample size (*n*) for each experimental group/condition, given as a discrete number and unit of measurement |
| x | | A statement on whether measurements were taken from distinct samples or whether the same sample was measured repeatedly |
| x | | The statistical test(s) used AND whether they are one- or two-sided<br>*Only common tests should be described solely by name; describe more complex techniques in the Methods section.* |
| x | | A description of all covariates tested |
| x | | A description of any assumptions or corrections, such as tests of normality and adjustment for multiple comparisons |
| x | | A full description of the statistical parameters including central tendency (e.g. means) or other basic estimates (e.g. regression coefficient) AND variation (e.g. standard deviation) or associated estimates of uncertainty (e.g. confidence intervals) |
| x | | For null hypothesis testing, the test statistic (e.g. *F*, *t*, *r*) with confidence intervals, effect sizes, degrees of freedom and *P* value noted<br>*Give P values as exact values whenever suitable.* |
| x | | For Bayesian analysis, information on the choice of priors and Markov chain Monte Carlo settings |
| x | | For hierarchical and complex designs, identification of the appropriate level for tests and full reporting of outcomes |
| x | | Estimates of effect sizes (e.g. Cohen's *d*, Pearson's *r*), indicating how they were calculated |

*Our web collection on statistics for biologists contains articles on many of the points above.*

## Software and code

Policy information about availability of computer code

| | |
|---|---|
| Data collection | without this part |
| Data analysis | without this part |

For manuscripts utilizing custom algorithms or software that are central to the research but not yet described in published literature, software must be made available to editors and reviewers. We strongly encourage code deposition in a community repository (e.g. GitHub). See the Nature Portfolio guidelines for submitting code & software for further information.

## Data

Policy information about availability of data

All manuscripts must include a data availability statement. This statement should provide the following information, where applicable:

- Accession codes, unique identifiers, or web links for publicly available datasets
- A description of any restrictions on data availability
- For clinical datasets or third party data, please ensure that the statement adheres to our policy

The data supporting the findings of this study are provided in this article and its Supplementary Information. Source data are available from the corresponding authors.

## Research involving human participants, their data, or biological material

Policy information about studies with human participants or human data. See also policy information about sex, gender (identity/presentation), and sexual orientation and race, ethnicity and racism.

| | |
|---|---|
| Reporting on sex and gender | without this part |
| Reporting on race, ethnicity, or other socially relevant groupings | without this part |
| Population characteristics | without this part |
| Recruitment | without this part |
| Ethics oversight | without this part |

Note that full information on the approval of the study protocol must also be provided in the manuscript.

# Field-specific reporting

Please select the one below that is the best fit for your research. If you are not sure, read the appropriate sections before making your selection.

☐ Life sciences ☐ Behavioural & social sciences ☐ Ecological, evolutionary & environmental sciences

For a reference copy of the document with all sections, see nature.com/documents/nr-reporting-summary-flat.pdf

# Life sciences study design

All studies must disclose on these points even when the disclosure is negative.

| | |
|---|---|
| Sample size | without this part |
| Data exclusions | without this part |
| Replication | without this part |
| Randomization | without this part |
| Blinding | without this part |

# Behavioural & social sciences study design

All studies must disclose on these points even when the disclosure is negative.

| | |
|---|---|
| Study description | without this part |
| Research sample | without this part |
| Sampling strategy | without this part |
| Data collection | without this part |
| Timing | without this part |
| Data exclusions | without this part |
| Non-participation | without this part |
| Randomization | without this part |

# Ecological, evolutionary & environmental sciences study design

All studies must disclose on these points even when the disclosure is negative.

| | |
|---|---|
| Study description | without this part |

| | |
|---|---|
| Research sample | without this part |
| Sampling strategy | without this part |
| Data collection | without this part |
| Timing and spatial scale | without this part |
| Data exclusions | without this part |
| Reproducibility | without this part |
| Randomization | without this part |
| Blinding | without this part |

Did the study involve field work? ☐ Yes ☒ No

# Reporting for specific materials, systems and methods

We require information from authors about some types of materials, experimental systems and methods used in many studies. Here, indicate whether each material, system or method listed is relevant to your study. If you are not sure if a list item applies to your research, read the appropriate section before selecting a response.

## Materials & experimental systems

| n/a | Involved in the study |
|---|---|
| ☒ | ☐ Antibodies |
| ☒ | ☐ Eukaryotic cell lines |
| ☒ | ☐ Palaeontology and archaeology |
| ☒ | ☐ Animals and other organisms |
| ☒ | ☐ Clinical data |
| ☒ | ☐ Dual use research of concern |
| ☒ | ☐ Plants |

## Methods

| n/a | Involved in the study |
|---|---|
| ☒ | ☐ ChIP-seq |
| ☒ | ☐ Flow cytometry |
| ☒ | ☐ MRI-based neuroimaging |

# Plants

| | |
|---|---|
| Seed stocks | without this part |
| Novel plant genotypes | without this part |
| Authentication | without this part |
